# The value of SPECT in the detection of stress injury to the pars interarticularis in patients with low back pain

**DOI:** 10.1186/1749-799X-5-13

**Published:** 2010-03-03

**Authors:** Katherine Zukotynski, Christine Curtis, Frederick D Grant, Lyle Micheli, S Ted Treves

**Affiliations:** 1 Division of Nuclear Medicine, Department of Imaging, Dana-Farber Cancer Institute, Boston, MA, USA; 2 Division of Sports Medicine, Department of Orthopedic Surgery, Children's Hospital Boston, Boston, MA, USA; 3 Division of Nuclear Medicine, Department of Radiology, Children's Hospital Boston, Boston, MA, USA; 4 Harvard Medical School, Boston, MA, USA

## Abstract

The medical cost associated with back pain in the United States is considerable and growing. Although the differential diagnosis of back pain is broad, epidemiological studies suggest a correlation between adult and adolescent complaints. Injury of the pars interarticularis is one of the most common identifiable causes of ongoing low back pain in adolescent athletes. It constitutes a spectrum of disease ranging from bone stress to spondylolysis and spondylolisthesis. Bone stress may be the earliest sign of disease. Repetitive bone stress causes bone remodeling and may result in spondylolysis, a non-displaced fracture of the pars interarticularis. A fracture of the pars interarticularis may ultimately become unstable leading to spondylolisthesis. Results in the literature support the use of bone scintigraphy to diagnose bone stress in patients with suspected spondylolysis. Single photon emission computed tomography (SPECT) provides more contrast than planar bone scintigraphy, increases the sensitivity and improves anatomic localization of skeletal lesions without exposing the patient to additional radiation. It also provides an opportunity for better correlation with other imaging modalities, when necessary. As such, the addition of SPECT to standard planar bone scintigraphy can result in a more accurate diagnosis and a better chance for efficient patient care. It is our expectation that by improving our ability to correctly diagnose bone stress in patients with suspected injury of the posterior elements, the long-term cost of managing this condition will be lowered.

## Introduction

The economic burden of back pain is estimated to be more than $90 billion per year in the United States [1,2]. Costs may be due to a variety of factors including primary care, diagnostic imaging, inpatient services, physical therapy and lost work productivity. Recent epidemiological studies suggest a correlation between adult and adolescent complaints [3,4].

The differential diagnosis for back pain is broad and includes degenerative disease, infection, inflammation, tumors and trauma [5-7]. Injury of the pars interarticularis is one of the most common identifiable causes of ongoing low back pain in adolescent athletes [6,8,9]. It constitutes a spectrum of disease from bone stress through spondylolysis and spondylolisthesis. Bone stress may be the earliest sign. It is most common at L5, which is particularly vulnerable to micro-trauma from repetitive flexion, extension or rotational forces. Repetitive bone stress may result in spondylolysis, a non-displaced fracture of the pars interarticularis. Ultimately spondylolisthesis, or slippage of one vertebral body on another, may occur.

## The Diagnosis and Treatment of Spondylolysis

Athletes comprise the majority of patients presenting with spondylolysis [10-12]. Sport specific maneuvers with repetitive twisting rotation and extension increase load on the spine, and may result in stress injury [13,14]. The most frequently presenting complaint is low back pain; either localized or diffuse [8,9,15]. In more severe cases, muscle spasms from difficulty in gait and posture may result.

The medical history should include duration of symptoms, modifying and alleviating factors, level and intensity of sport participation as well as changes in muscle, bowel and bladder function. Physical examination involves inspection and palpation of the spine as well as examination of range of motion [9]. Inspection of the spine may reveal hyperlordosis. Palpation for tenderness is useful to identify area(s) of stress, fracture, or slippage [9]. Range of motion is frequently more compromised and painful in extension. The stork test may reveal pain on the contralateral side when standing on one leg. While this test is not specific for pars stress injury, it is highly suggestive of some type of derangement of the posterior elements of the spine [16]. Imaging studies used to evaluate patients with low back pain include: radiographs, bone scintigraphy, computed tomography (CT) and magnetic resonance imaging (MRI).

**Figure 1:**
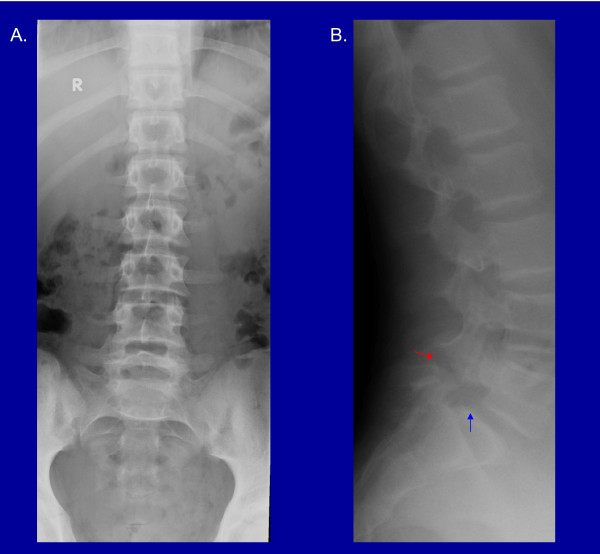
**Radiographic findings in a patient with L5 pars interarticularis fracture and mild L5 on S1 spondylolisthesis: AP (A) and lateral (B) images**. [Red arrow points to the fracture and blue arrow points to spondylolisthesis of L5 on S1].

Radiographs of the spine have limited sensitivity compared with other imaging modalities in detecting bone stress and acute spondylolysis. Furthermore, radiographic defects of the pars interarticularis may not be symptomatic [17,18]. Figure 1 illustrates the radiographic appearance of a long standing pars interarticularis defect.

**Figure 2:**
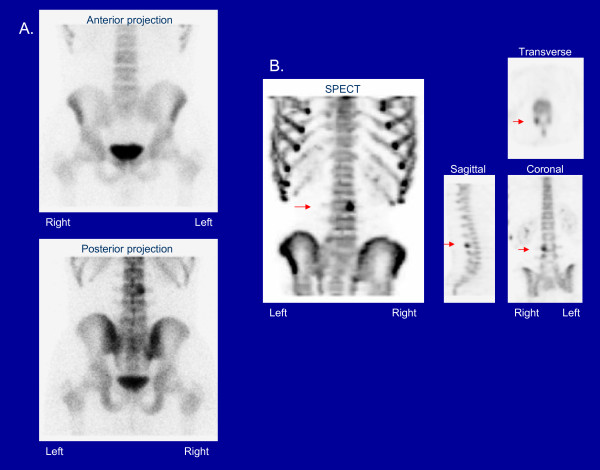
**Scintigraphic findings in a patient with right L3 pars stress on planar bone scintigraphy (A) and on SPECT (B)**. [Red arrows point to the scintigraphic abnormality on SPECT].

**Figure 3:**
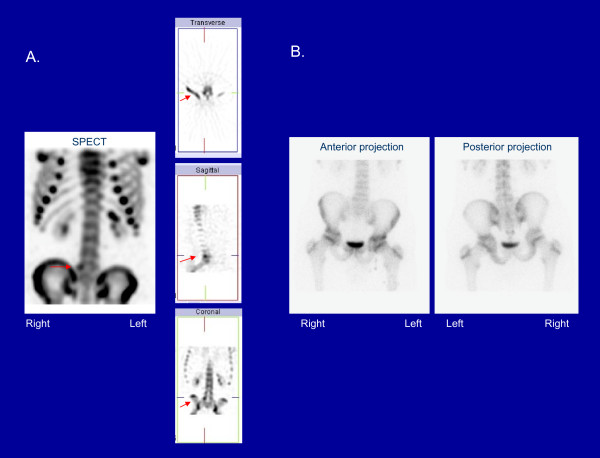
**Scintigraphic findings in a patient with right L5 pars stress on SPECT (A), not seen on planar bone scintigraphy (B)**. [Red arrows point to the scintigraphic abnormality].

Bone scintigraphy is very sensitive for the detection of bone stress. Repetitive stress causes local bone remodeling and abnormal uptake of scintigraphic tracer. Single photon emission computed tomography (SPECT) has 10-20 times more contrast than planar bone scintigraphy and is more sensitive than radiography and planar bone scans. Furthermore, scintigraphic abnormalities have been found to correlate with painful lesions of the pars interarticularis [18-21]. The diagnosis of spondylolisthesis is not made with scintigraphy. Once spondylolisthesis develops, bone stress may be absent at the site of spondylolysis. However, in this case, bone remodeling and tracer uptake may occur at the pars interarticularis immediately above or below the level of fracture. Figure 2 illustrates stress of the pars interarticularis on bone scintigraphy. Figure 3 presents an example where pars stress is identified on SPECT but not on planar bone scintigraphy.

**Figure 4:**
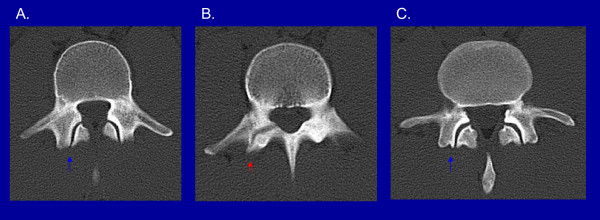
**CT findings in a patient with pars interarticularis fracture: Normal facet joint below fracture (A), right L3 pars interarticularis fracture (B), normal facet joint above fracture (C)**. [red arrow points to the fracture and blue arrows point to normal facet joints].

CT demonstrates detailed osseous morphology, is more specific than bone scintigraphy and may predict the probability of ultimate bone healing [22,23]. However, CT of the spine results in higher ionizing radiation exposure compared to bone scintigraphy [24]. Furthermore, there are reports in the literature of a normal spine CT in patients with abnormalities on planar bone scintigraphy and SPECT [16,25]. This may be explained by the fact that tracer uptake in the region of the pars interarticularis on scintigraphic studies corresponds to bone stress. If this stress has not yet resulted in a fracture, changes may not be visible on CT. The identification of patients with this pattern of scintigraphic findings is particularly important as these patients may have the best chance of healing with early treatment [6]. Figure 4 shows a fracture of the pars interarticularis on CT.

MRI is not as sensitive as SPECT for identifying bone stress of the pars interarticularis and does not delineate bony detail to the same extent as CT [16,25]. MRI is, however, attractive as an imaging modality that does not involve ionizing radiation and that is excellent in identifying alternate pathology including bone edema or abnormalities of the soft tissues, disk and spinal cord.

In general, when bone stress or spondylolysis is suspected, bone scintigraphy with SPECT is recommended. If SPECT demonstrates a pars lesion, a thin-cut CT (1 mm axial sequence) through the area of abnormality on SPECT, is recommended to confirm the diagnosis and stage the lesion. If SPECT is negative, pars stress is unlikely to be the cause of the low back pain and MRI may be helpful in identifying other causes of back pain [6,26].

Complete bony union offers the best long term prognosis. Some patients attain a fibrous union and are consequently able to return to prior activity, with favorable short-term prognoses. Treatment often includes rest from aggravating activities, non steroidal anti-inflammatory medication, bracing and physical therapy emphasizing hamstring stretching and core strengthening. The length of activity restriction, use of bracing and type of rehabilitation programs varies, reflecting a lack of consensus among practitioners. In recalcitrant cases, electrical stimulation may be added [9,27,28]. Prompt treatment of patients with early pars stress has been shown to result in more predictable symptom relief and less likelihood of progression to spondylolisthesis [29-31]. Surgery is reserved for patients who do not respond to conservative management (approximately 5%), have progressive spondylolisthesis, intractable pain or neurological deficits [18].

## The Utility of SPECT over Planar Bone Scintigraphy in the Evaluation of Back Pain

Studies have consistently demonstrated that SPECT is more sensitive than planar bone scintigraphy to identify skeletal lesions [31-33]. Collier et al. compared planar bone scintigraphy and SPECT in 19 adults with radiographic evidence of spondylolysis and/or spondylolisthesis and found that SPECT was more sensitive in identifying sites of "painful" pars interarticularis defects and that SPECT allowed more accurate localization of the defect [19]. In a long-term follow-up study, Bellah et al. reviewed findings on planar and SPECT bone scintigraphy in 162 patients aged 6-32 years with symptoms of low back pain potentially related to stress injury of the pars interarticularis. SPECT showed an abnormal focus of radiotracer uptake in the lumbar spine in 71 patients (44%). All abnormalities detected on planar bone scintigraphy were detected with SPECT. An abnormality was identified in 39 patients (24%) on SPECT alone [20]. Even-Sapir et al. demonstrated SPECT was more sensitive and specific than planar bone scintigraphy in the detection of bone metastasis in a prospective study of 44 patients with prostate cancer [32]. Strobel et al. found that lesion visibility as well as the ability to determine a specific diagnosis was significantly better for SPECT than with planar bone scintigraphy [33].

We conducted an internal review of all patients with low back pain or suspected spondylolysis referred to the Division of Nuclear Medicine at Children's Hospital, Boston for skeletal scintigraphy between October 2005 and September 2006. Of 115 identified patients undergoing skeletal SPECT and planar scintigraphy, SPECT identified an abnormal focus of increased tracer uptake in the pars interarticularis in 42 patients (37%). All abnormalities detected on planar bone scintigraphy were also detected with SPECT. Planar bone scintigraphy identified an abnormal focus of tracer uptake in the pars interarticularis in 19 patients (17%). SPECT identified additional sites of pars stress in 5 of the 19 patients with pars stress suggested on planar bone scintigraphy (26%).

In general, SPECT increases contrast and improves anatomic localization in comparison to planar scintigraphy [34]. In SPECT, images are acquired in multiple projections with the gamma scintillation camera traversing an axial orbit about the patient. Filtered back projection (FBP) or an iterative reconstruction algorithm such as OSEM (ordered subsets expectation maximization) is then used to create a cross-sectional image. The cross-sectional image is a two-dimensional representation of a slice through the patient that would project onto a single dimension on a planar bone scan. In addition, SPECT images may be displayed as a 3D representation using a volume rendered display to provide better spatial orientation. Maeseneer et al. illustrated how patterns of tracer uptake in the spine on SPECT suggested specific pathology [35]. Degenerative disk disease might show increased tracer uptake centered about the disk space. Pars interarticularis stress might show tracer uptake in the expected location of the pars interarticularis and metastatic disease is more likely to involve the vertebral body with extension to the pedicle [35,36]. Ultimately, SPECT may be fused with CT, if needed, to help add specificity to the findings.

## Conclusions

The economic burden of back pain is significant and growing. Epidemiological studies suggest a correlation between adult and adolescent complaints. Pars interarticularis injury, a spectrum of disease ranging from bone stress to spondylolysis and spondylolisthesis, is the most common identifiable cause of ongoing low back pain in adolescent athletes.

In the current era of multi-modality imaging, radiographs, skeletal scintigraphy, CT and MRI all play an important role in imaging patients with back pain. Planar bone scintigraphy has a long history in the diagnosis of patients with suspected injury of the pars interarticularis because it is more sensitive than radiographs for localizing the site of bone stress and because CT of the spine is associated with significant ionizing radiation. The addition of SPECT to planar skeletal scintigraphy increases sensitivity and improves disease localization without exposing the patient to additional radiation. SPECT can also identify early pars stress prior to the development of osseous change detectable with CT. As such, incorporation of SPECT into the standard planar bone scintigraphy routine should lead to a more accurate initial diagnosis. It is our hypothesis that by improving our ability to promptly diagnose patients with suspected injury of the pars interarticularis, the patients will be better served and the long-term cost of management can be lowered.

## List of abbreviations used

SPECT: Single photon emission computed tomography; CT: Computed tomography; MRI: Magnetic resonance imaging.

## Competing interests

The authors declare that they have no competing interests.

## Authors' Information

Katherine Zukotynski is an Instructor in Radiology at Harvard Medical School and Christine Curtis is Team Leader in Clinical Research at the Children's Hospital Boston. Frederick D Grant is an Instructor in Radiology  at Harvard Medical School. Lyle Micheli is a Professor of Orthopedic Surgery at Harvard Medical School. Ted Treves is a Professor of Radiology at Harvard Medical School.  

## Authors' contributions

All of the authors have read and approved the final manuscript.

## References

[B1] Dagenais S Caro J Haldeman S A systematic review of low back pain cost of illnessstudies in the United States and internationally The Spine Journal 2008 8 8 20 10.1016/j.spinee.2007.10.005 18164449

[B2] Luo X Pietrobon R Sun S Liu G Hey L Estimates and patterns of direct health care expenditures among individuals with back pain in the United States Spine 2004 29 1 79 86 10.1097/01.BRS.0000105527.13866.0F 14699281

[B3] Kim H Green D Adolescent back pain Current Opinion in Pediatrics 2008 20 37 45 10.1097/MOP.0b013e3282f357fe 18197037

[B4] Leboeuf-Yde C Kyvik K At what age does low back pain become a common problem?: A study of 29,424 individuals aged 12-41 years Spine 1998 23 228 234 10.1097/00007632-199801150-00015 9474731

[B5] Bernstein R Cozen H Evaluation of back pain in children and adolescents American Family Physician 2007 76 11 1669 1676 18092709

[B6] Standaert C Herring S Expert opinion and controversies in sports andmusculoskeletal medicine: The diagnosis and treatment of spondylolysis in adolescent athletes Archives of Physical Medicine and Rehabilitation 2007 88 537 540 10.1016/j.apmr.2007.01.007 17398258

[B7] Gregory P Batt M Kerslake R Webb J Single photon emission computerized tomography and reverse gantry computerized tomography findings in patients with back pain investigated for spondylolysis Clinical Journal of Sport Medicine 2005 15 2 79 86 10.1097/01.jsm.0000152710.82225.3d 15782051

[B8] Micheli LJ Wood R Back pain in young athletes. Significant differences from adults in causes and patterns Archives of Pediatrics and Adolescent Medicine 1995 149 15 18 7827653 10.1001/archpedi.1995.02170130017004

[B9] Micheli LJ Curtis C Stress fractures in the spine and sacrum Clinics in Sports Medicine 2006 25 1 75 88 10.1016/j.csm.2005.08.001 16324975

[B10] Micheli LJ Low back pain in the adolescent: differential diagnosis The American Journal of Sports Medicine 1979 7 362 364 10.1177/036354657900700613 159627

[B11] Micheli LJ Back injuries in dancers Clinical Journal of Sports Medicine 1983 2 3 473 484 6228309

[B12] d'Hemecourt PA Zurakowski D Kriemler S Micheli LJ Spondylolysis: returning the athlete to sports participation with brace treatment Orthopedics 2002 25 6 653 657 12083575 10.3928/0147-7447-20020601-15

[B13] Weir MR Smith DS Stress reaction of the pars interarticularis leading to spondylolysis. A cause of adolescent low back pain Journal of Adolescent Health Care 1989 10 6 573 577 10.1016/0197-0070(89)90029-6 2532632

[B14] Beutler WJ Frederickson BE Murtland A Sweeney CA Grant WD Baker D The natural history of spondylolysis and spondylolisthesis: 45 year follow-up evaluation Spine 2003 28 10 1027 1035 10.1097/00007632-200305150-00014 12768144

[B15] Tallarico RA Madom IA Palumbo MA Spondylolysis and spondylolisthesis in the athlete Sports Medicine and Arthroscopy Review 2008 16 1 32 38 10.1097/JSA.0b013e318163be50 18277260

[B16] Masci L Pike J Malara F Phillips B Bennell K Brukner P Use of the one-legged hyperextension test and magnetic resonance imaging in the diagnosis of active spondylolysis British Journal of Sports Medicine 2006 40 940 946 10.1136/bjsm.2006.030023 16980534 PMC2465027

[B17] Pennell R Maurer A Bonakdarpour A Stress injuries of the pars interarticularis: radiologic classification and indications for scintigraphy American Journal of Roentgenology 1985 145 763 766 3875996 10.2214/ajr.145.4.763

[B18] Standaert C Herring S Spondylolysis: a critical review British Journal of Sports Medicine 2000 34 415 422 10.1136/bjsm.34.6.415 11131228 PMC1724260

[B19] Collier B Johnson R Carrera G Meyer G Schwab J Flatley T Painful spondylolysis or spondylolisthesis studied by radiography and single-photon-emission computed tomography Radiology 1985 154 207 211 3155479 10.1148/radiology.154.1.3155479

[B20] Bellah R Summerville D Treves S Micheli L Low-back pain in adolescent athletes: detection of stress injury to the pars interarticularis with SPECT Muskuloskeletal Radiology 1991 180 509 512 10.1148/radiology.180.2.1829845 1829845

[B21] Harvey C Richenberg J Saifuddin A Wolman R Pictoral review: The radiological investigation of lumbar spondylolysis Clinical Radiology 1998 53 723 728 10.1016/S0009-9260(98)80313-9 9817088

[B22] Congeni J McCulloch J Swanson K Lumbar Spondylolysis: A study of natural progression in athletes The American Journal of Sports Medicine 1997 25 2 248 253 10.1177/036354659702500220 9079183

[B23] Morita T Ikata T Katoh S Miyake R Lumbar spondylolysis in children and adolescents Journal of Bone and Joint Surgery 1995 77 4 620 625 7615609

[B24] Brenner D Hall E Computed Tomography - An Increasing Source of Radiation Exposure The New England Journal of Medicine 2007 357 2277 2284 10.1056/NEJMra072149 18046031

[B25] Campbell R Grainger A Hide I Papastefanou S Greenough C Juvenile spondylolysis: a comparative analysis of CT, SPECT, and MRI Skeletal Radiology 2005 34 63 73 10.1007/s00256-004-0878-3 15668821

[B26] Gregory P Batt M Kerslake R Scammell B Webb J The value of combining single photon emission computerized tomography and computerized tomography in the investigation of spondylolysis European Spine Journal 2004 13 503 509 10.1007/s00586-004-0696-2 15118897 PMC3476601

[B27] Fellander-Tsai L Micheli LJ Treatment of spondylolysis with extreme electrical stimulation and bracing in adolescent athletes: a report of 2 cases Clinical Journal of Sport Medicine 1998 8 3 232 234 10.1097/00042752-199807000-00012 9762484

[B28] Pettine KA Salib RM Walker SG External electrical stimulation and bracing for treatment of spondylolysis. A case report Spine 1993 18 4 436 439 8470003

[B29] Motley G Nyland J Jacobs J Caborn D The pars interarticularis stress reaction, spondylolysis, and spondylolisthesis progression Journal of Athletic Training 1998 33 4 351 358 16558534 PMC1320587

[B30] Takemitsu M Rassi G Woratanarat P Shah S Low back pain in pediatric athletes with unilateral tracer uptake at the pars interarticularis on single photon emission computed tomography Spine 2006 31 8 909 914 10.1097/01.brs.0000209308.19642.96 16622380

[B31] Anderson K Sarwark J Conway J Logue S Schafer M Quantitative assessment with SPECT imaging of stress injuries of the pars interarticularis and response to bracing Journal of Pediatric Orthopaedics 2000 20 1 28 33 10.1097/00004694-200001000-00007 10641684

[B32] Even-Sapir E Metser U Mishani E Lievshitz G Lerman H Leibovitch I The detection of bone metastasis in patients with high-risk prostate cancer: 99mTc-MDP planar bone scintigraphy, single- and multi-field-of-view SPECT, 18F-Fluoride PET, and 18F-Fluoride PET/CT The Journal of Nuclear Medicine 2006 47 2 287 297 16455635

[B33] Strobel K Burger C Seifert B Husarik D Soyka J Hany T Characterization of focal bone lesions in the axial skeleton: performance of planar bone scintigraphy compared with SPECT and SPECT fused with CT American Journal of Roentgenology 2007 188 W467 474 10.2214/AJR.06.1215 17449746

[B34] Sarikaya I Sarikaya A Holder L The role of single photon emission computed tomography in bone imaging Seminars in Nuclear Medicine 2001 31 1 3 16 10.1053/snuc.2001.18736 11200204

[B35] Maeseneer M Lenchik L Everaert H Marcelis S Bossuyt A Osteaux M Beeckman P Evaluation of lower back pain with bone scintigraphy and SPECT Radiographics 1999 19 901 912 10464798 10.1148/radiographics.19.4.g99jl03901

[B36] Reinartz P Schaffeldt J Sabri O Zimny M Nowak B Otswald E Cremerius U Udalrich B Benign versus malignant osseous lesions in the lumbar vertebrae: differentiation by means of bone SPET European Journal of Nuclear Medicine 2000 27 6 721 726 10.1007/s002590050568 10901460

